# In defense of pleasure: We need to rethink food reward and obesity

**DOI:** 10.1371/journal.pbio.3003497

**Published:** 2025-11-13

**Authors:** Justin J. Sung, Dana M. Small

**Affiliations:** 1 Department of Neurology and Neurosurgery, Medicine and Psychology, McGill University, Montreal, Québec, Canada; 2 Research Institute of the McGill University Hospital Centre, Montreal, Québec, Canada; 3 McConnell Brain Imaging Centre, Montreal, Québec, Canada; 4 Modern Diet and Physiology Research Centre, Montreal, Québec, Canada

## Abstract

Is overeating driven by hedonism? This Perspective argues that the pleasure of eating food is not a driver of the obesity epidemic, but rather the regular consumption of an unhealthy diet blunts sensitivity to interoceptive signals that drive food reward.

## Teaser

Is overeating driven by hedonism? We argue that the pleasure of eating food is not a driver of the obesity epidemic, but rather the regular consumption of an unhealthy diet blunts sensitivity to interoceptive signals that drive food reward.

All organisms need to acquire energy to sustain life. To optimize this behavior, the values of foods are learned and updated based on experienced physiological benefit. For example, illness following food consumption leads to the formation of a conditioned taste aversion so that the food can be avoided in the future. By contrast, when consumption leads to nutrient and energy availability, a conditioned preference (flavor nutrient learning) is formed, and the value of food cues and flavors increases, motivating acquisition and intake at a level that equates to physiological value. Importantly, whereas conditioned taste aversion is associated with a host of unpleasant conscious sensations, nutrient and energy sensing is largely subliminal and interoceptive, being triggered when nutrients are detected in the upper intestine or energy use is detected in the hepatoportal vein. These subliminal neural signals are carried by the spinal and vagus nerves to the central nervous system to release dopamine and support learning and value updating, acting as a primary reinforcer [1].

Critically, although such subliminal reinforcers can influence conscious perceptions (for example, by increasing liking), they can also cause conditioning and influence behavior and intake independently of conscious “hedonic” correlates. For example, in humans, such post-oral interoceptive reinforcers condition increased intake [2] and responses in the nucleus accumbens and hypothalamus to flavors [3], and they do so completely independently of the effects of conditioning on the conscious perception of liking. This demonstrates a dissociation between circuits coding for what we refer to as “high road” (e.g., delicious flavors) versus “low road” (subliminal post-oral) reinforcers [1]. Moreover, despite being subliminal, these low road signals are both necessary and sufficient to sustain food reward and therefore act as the critical unconditioned reinforcer behind food reward [4]. By contrast, the conscious sensation of food, including encounters with food cues and delicious flavors, are conditioned secondary reinforcers that are neither necessary nor sufficient to sustain reward in the absence of post-oral interoceptive reinforcement [4].

Although both high road and low road pathways and their integration are critical for adaptive food decisions, the bulk of evidence indicates that high road hedonic signals are not to blame for the obesity epidemic. Nevertheless, researchers continue to promote the idea that hyperpalatable foods lead to obesity and to target “hedonic” systems in the search for new therapeutic targets. We believe that this framework is problematic and would urge a shift of focus towards interoceptive reward signals and gut–brain learning, rather than hedonic behaviors.

If hedonic (i.e., pleasurable) signals are driving the obesity epidemic, then foods linked with the obesity epidemic should be more pleasurable, and those who experience more pleasure from these foods should have higher body mass indices. Following the same logic, the glucagon-like peptide-1 (GLP-1) agonists that are so effective at reducing food intake should blunt the pleasure derived from food. However, although ultraprocessed foods (UPFs) have been linked to the obesity epidemic and found to promote overconsumption compared to minimally processed foods, the UPFs used in these studies were not rated as more liked, and therefore must promote food intake by some other means [5]. Likewise, although increased sugar intake is related to increased rates of obesity, extreme sweet-likers have greater lean mass, but not fat mass. They also have higher interoceptive accuracy for both generic (heartbeats) and gut-based (gastric satiation and fullness) signals [6]. This suggests that increased liking reflects a sensitivity to higher metabolic requirements rather than representing a driver of obesity. Furthermore, GLP-1 agonists do not appear to reduce food intake by reducing food liking [7]. Therefore, it is unlikely that eating for pleasure drives the obesity epidemic.

Instead, there is strong evidence from animal models that habitual consumption of unhealthy diets rewires both hypothalamic and midbrain-striatal circuits to promote overeating and obesity risk by reducing sensing of nutrients by the digestive tract, thereby decreasing the value of less energy-dense foods, producing compulsive and impulsive behavior, impairing memory, and reducing taste perception [8]. Many of these effects are observed in the absence of weight gain or metabolic change [9]. While experimental use of diets analogous to those used in animals is challenging in humans, studies that use mild, naturalistic dietary interventions do show neurobehavioral alterations that are similar to what has been reported in rodent models. For example, healthy weight individuals randomized to consume a high-fat and high-sugar versus an equicaloric low-fat and low-sugar snack daily for 8 weeks show decreased liking of a low-fat test food, despite no differences in weight gain or metabolic measures [10]. In animals, a high-fat diet (HFD)-induced decreased preference for low-fat foods can be recovered by increasing post-oral signaling [9], suggesting that the decreased preference for low-fat food observed in humans [10] may reflect reduced post-oral sensitivity to fat. Accordingly, nasogastric delivery of lipids to humans with healthy weight, but not to those with obesity, decreases striatal response, an effect that does not reverse following a 10% reduction in body weight [11]. Exposure to the high-fat/high-sugar versus low-fat/low-sugar diet also revealed enhanced neural responses to food cues and enhanced sensory association learning. This is consistent with a large literature linking food cue reactivity in humans to risk for weight gain [12], as well as with animal studies showing that exposure to habitual HFD increases impulsive responding (an effect that is associated with decreases in dopamine receptor subtype 2 signaling) and can do so without increases in body weight [13].

Collectively, the findings from human and animal studies converge to suggest that it is decreased sensitivity to the subliminal interoceptive signals that are generated during nutrient digestion, coupled with increased stimulus-driven intake, that drives overeating, rather than a heightened hedonic signal. This conclusion is consistent with the incentive-sensitization theory of addiction [14]. In the case of feeding, it also highlights a critical contribution of interoceptive sensitivity. However, much remains unknown regarding this gut–brain system. Characterizing the trajectory, dose, and reversibility of diet-induced neuroplasticity will better inform the development of effective treatments that restore signal fidelity, the creation of regulations on the food industry that limit obesogenic foods, and the design of dietary guidelines that protect interoceptive function. This reorientation from hedonic pleasure to gut–brain communication represents a necessary next step in addressing the global obesity epidemic.

## Abbreviations

**:**
GLP-1: glucagon-like peptide-1
HFD: high-fat diet
UPFs: ultraprocessed foods

## References

[1] de Araujo IE, Schatzker M, Small DM. Rethinking food reward. Annu Rev Psychol. 2020;71:139–64. doi: 10.1146/annurev-psych-122216-011643 31561741

[2] Ribeiro G, Fernandes AB, Oliveira FPM, Duarte JS, Oliveira M, Limbert C, et al. Postingestive reward acts through behavioral reinforcement and is conserved in obesity and after bariatric surgery. PLoS Biol. 2024;22(12):e3002936. doi: 10.1371/journal.pbio.3002936 39689052 PMC11651594

[3] de Araujo IE, Lin T, Veldhuizen MG, Small DM. Metabolic regulation of brain response to food cues. Curr Biol. 2013;23(10):878–83. doi: 10.1016/j.cub.2013.04.001 23643837 PMC3767438

[4] de Araujo IE, Oliveira-Maia AJ, Sotnikova TD, Gainetdinov RR, Caron MG, Nicolelis MAL, et al. Food reward in the absence of taste receptor signaling. Neuron. 2008;57(6):930–41. doi: 10.1016/j.neuron.2008.01.032 18367093

[5] Hall KD, Ayuketah A, Brychta R, Cai H, Cassimatis T, Chen KY, et al. Ultra-processed diets cause excess calorie intake and weight gain: an inpatient randomized controlled trial of ad libitum food intake. Cell Metab. 2019;30(1):67-77.e3. doi: 10.1016/j.cmet.2019.05.008 31105044 PMC7946062

[6] Iatridi V, Armitage RM, Yeomans MR, Hayes JE. Effects of sweet-liking on body composition depend on age and lifestyle: a challenge to the simple sweet-liking-obesity hypothesis. Nutrients. 2020;12(9):2702. doi: 10.3390/nu12092702 32899675 PMC7551752

[7] Coppin G, Muñoz Tord D, Pool ER, Locatelli L, Achaibou A, Erdemli A, et al. A randomized controlled trial investigating the effect of liraglutide on self-reported liking and neural responses to food stimuli in participants with obesity. Int J Obes (Lond). 2023;47(12):1224–31. doi: 10.1038/s41366-023-01370-w 37626125 PMC10663148

[8] Ferrario CR, Münzberg-Gruening H, Rinaman L, Betley JN, Borgland SL, Dus M, et al. Obesity- and diet-induced plasticity in systems that control eating and energy balance. Obesity (Silver Spring). 2024;32(8):1425–40. doi: 10.1002/oby.24060 39010249 PMC11269035

[9] Tellez LA, Medina S, Han W, Ferreira JG, Licona-Limón P, Ren X, et al. A gut lipid messenger links excess dietary fat to dopamine deficiency. Science. 2013;341(6147):800–2. doi: 10.1126/science.1239275 23950538

[10] Edwin Thanarajah S, DiFeliceantonio AG, Albus K, Kuzmanovic B, Rigoux L, Iglesias S, et al. Habitual daily intake of a sweet and fatty snack modulates reward processing in humans. Cell Metab. 2023;35(4):571-584.e6. doi: 10.1016/j.cmet.2023.02.015 36958330

[11] van Galen KA, Schrantee A, Ter Horst KW, la Fleur SE, Booij J, Constable RT, et al. Brain responses to nutrients are severely impaired and not reversed by weight loss in humans with obesity: a randomized crossover study. Nat Metab. 2023;5(6):1059–72. doi: 10.1038/s42255-023-00816-9 37308722

[12] Boswell RG, Kober H. Food cue reactivity and craving predict eating and weight gain: a meta-analytic review. Obes Rev. 2016;17(2):159–77. doi: 10.1111/obr.12354 26644270 PMC6042864

[13] Adams WK, Sussman JL, Kaur S, D’souza AM, Kieffer TJ, Winstanley CA. Long-term, calorie-restricted intake of a high-fat diet in rats reduces impulse control and ventral striatal D2 receptor signalling—two markers of addiction vulnerability. Eur J Neurosci. 2015;42(12):3095–104. doi: 10.1111/ejn.13117 26527415

[14] Robinson TE, Berridge KC. The neural basis of drug craving: an incentive-sensitization theory of addiction. Brain Res Brain Res Rev. 1993;18(3):247–91. doi: 10.1016/0165-0173(93)90013-p 8401595

